# The experience of a charity in translating the results of basic research to therapies for patients

**DOI:** 10.1186/1750-1172-9-S1-O20

**Published:** 2014-11-11

**Authors:** Lucia Monaco

**Affiliations:** 1 Fondazione Telethon, Milan, Italy

## 

Rare diseases represent a relevant societal challenge that calls different players into action, with the aim of providing a diagnosis to patients, understanding the disease pathophysiology and, most importantly, developing treatments and therapies to effectively improve life quality and expectancy.

Biomedical research charities play an important role in the fight against rare diseases, as they are driven by a strong patient need and are focused on diseases otherwise poorly supported by public or private funders. Since 1990, the Telethon Foundation has supported research on genetic diseases, most of which are rare, through intramural and extramural investments in Italy based on strict, excellence-driven fund allocation criteria.

Although Telethon-funded research still relies on a strong base of fundamental studies aimed at disclosing the pathophysiology of genetic diseases, it has progressively shifted towards preclinical and clinical studies, today standing at 50% of Telethon’s investments. In particular, the considerable expertise on gene therapy built at the Telethon Institute for Gene Therapy (TIGET), a joint initiative with the San Raffaele Hospital in Milan, has led to the first safe and effective gene therapy for a genetic disease, the severe immunodeficiency ADA-SCID [1]. This goal was achieved with continued support by the charity, which included creating a dedicated clinical trial office for regulatory support and training of specialized staff and bearing the cost and risks of the production of the therapeutic vector according to good manufacturing practices.

Finally, making this therapy available to patients required the skills and resources of a pharmaceutical company; dealing with an ultra-rare disease such as ADA-SCID presented a challenge that was met by GlaxoSmithKline (GSK). In 2010, Telethon/San Raffaele signed an agreement with GSK, including a license for the development of the ADA-SCID retroviral gene therapy and a collaboration program for six more genetic diseases based on a lentiviral gene therapy platform. TIGET has recently obtained clinical proof of concept for the first two diseases in the pipeline: metachromatic leukodystrophy and Wiskott-Aldrich syndrome [[3],4]. Meanwhile, GSK is progressing towards registration of the ADA-SCID therapy, a process still entailing Telethon’s direct involvement, besides close collaboration with TIGET.

The partnership between Telethon and GSK illustrates a novel collaborative model whereby a charity fulfills its traditional role as a funder and also acts as a driver for translating research results into the clinic and promoting transition to the industrial development, to the final benefit of patients.
